# Visual subcircuit-specific dysfunction and input-specific mispatterning in the superior colliculus of fragile X mice

**DOI:** 10.1186/s11689-018-9241-1

**Published:** 2018-06-28

**Authors:** Rachel B. Kay, Nicole A. Gabreski, Jason W. Triplett

**Affiliations:** 1 Center for Neuroscience Research, Children’s National Medical Center, Washington, DC, USA; 2 Departments of Pediatrics and Pharmacology & Physiology, The George Washington University School of Medicine and Health Sciences, Washington, DC, USA

**Keywords:** Receptive field, Direction selective, Axis selective

## Abstract

**Background:**

Sensory processing deficits are frequently co-morbid with neurodevelopmental disorders. For example, patients with fragile X syndrome (FXS), caused by a silencing of the *FMR1* gene, exhibit impairments in visual function specific to the dorsal system, which processes motion information. However, the developmental and circuit mechanisms underlying this deficit remain unclear. Recently, the superior colliculus (SC), a midbrain structure regulating head and eye movements, has emerged as a model for dissecting visual circuit development and function. Previous studies have demonstrated a critical role for activity-dependent processes in the development of visual circuitry in the SC. Based on the known role of the *FMR1* gene product in activity-dependent synaptic plasticity, we explored the function and organization of visual circuits in the SC of a mouse model of FXS (*Fmr1*^*−/y*^).

**Methods:**

We utilized in vivo extracellular electrophysiology in combination with computer-controlled visual stimuli to determine the receptive field properties of visual neurons in the SC of control and *Fmr1*^*−/y*^ mice. In addition, we utilized anatomical tracing methods to assess the organization of visual inputs to the SC and along the retinogeniculocortical pathway.

**Results:**

Receptive fields of visual neurons in the SC of *Fmr1*^*−/y*^ mice were significantly larger than those found in control animals, though their shape and structure were unaffected. Further, selectivity for direction of movement was decreased, while selectivity to axis of movement was unchanged. Interestingly, axis-selective (AS) neurons exhibited a specific hyperexcitability in comparison to AS neurons in control SC and to direction-selective (DS) neurons in both control and *Fmr1*^*−/y*^ SC. Anatomical tracings revealed that retinocollicular, retinogeniculate, and geniculocortical projections were normally organized in the absence of *Fmr1*. However, projections from primary visual cortex (V1) to the SC were poorly refined.

**Conclusions:**

*Fmr1* is required for the proper development of visual circuit organization and function in the SC. We find that visual dysfunction is heterogeneously manifested in a subcircuit-specific manner in *Fmr1*^*−/y*^ mice, consistent with previous studies in human FXS patients. Further, we show a specific alteration of inputs to the SC from V1, but not the retina. Together, these data suggest that *Fmr1* may function in distinct ways during the development of different visual subcircuits.

## Background

Sensory processing disorders occur frequently in the pediatric population, affecting as many as 1 in 20 children at some point in their development [1, 2]. The prevalence of sensory dysfunction is even higher among those with a neurodevelopmental disorder, such as autism spectrum disorders (ASD) [3] and fragile X syndrome (FXS) [4]. Since sensory deficits may amplify symptoms and interfere with therapies [5], they present an attractive target for intervention that may have a broad and multiplicative impact. However, while sensory dysfunction is a hallmark feature of many neurodevelopmental disorders, there is a gap in our understanding of the underlying circuit-level causes.

FXS, an X-linked neurodevelopmental disorder, is the most common hereditable form of intellectual disability and the most prevalent single gene cause of ASD. It is caused by the silencing of the *FMR1* gene, resulting in reduced expression of the gene product, fragile X mental retardation protein (FMRP), an RNA-binding protein. In the brain, FMRP is a critical player in activity-dependent synaptic plasticity, and thus circuit formation and function [6]. Patients with FXS exhibit a wide range of sensory dysfunctions that manifest in multiple modalities, including vision, audition, and somatosensation [7]. In the visual domain, patients show impaired magnocellular pathway function, which processes information about stimulus movement, while visual form detection is unaffected [8]. Interestingly, function in this visual domain is also altered in *FMR1* premutation carriers [9] and may be correlated with the level of FMRP expression in healthy individuals [10]. Despite this, little is known about how loss of FMRP expression affects the function of visual neurons in the brain.

In recent years, the mouse superior colliculus (SC) has emerged as an attractive model to understand sensory circuit development, organization, and function [11]. The SC processes visual, somatosensory and auditory information to regulate goal-directed head and eye movements [12]. Of these modalities in the SC, the development of visual circuitry is most well understood and relies on a combination of molecular cues, axon-axon competition, and activity-dependent processes [13]. In addition, our understanding of visual function and circuit wiring in the SC has advanced substantially due to clever application of advanced genetic, electrophysiological, imaging, and viral tracing techniques [14–18].

Based on the critical role of FMRP in activity-dependent synaptic plasticity and the dependence of visual circuit development in the SC on activity-dependent processes, we tested the hypothesis that visual circuit function and organization in the SC requires FMRP expression. To do so, we determined the receptive field properties of visual neurons in the SC of a mouse model of FXS in which the *Fmr1* gene is knocked out (*Fmr1*^*−/y*^) [19]. We show that visual receptive fields are increased in size, but retain normal shape and substructure. Further, we found that selectivity for direction of movement is decreased in the SC of *Fmr1*^*−/y*^ mice, but neurons tuned to the movement of oriented bars along their orthogonal axes are unchanged. Interestingly, axis-selective (AS) neurons were hyperexcitable, despite retaining their tuning properties. We also found that the terminal areas of inputs to the SC from the primary visual cortex (V1) are enlarged in *Fmr1* knockouts, while those from the retina are unaffected. Together, these data suggest that FMRP is required for the proper development of visual circuit organization and function in the SC and, further, that FMRP may function in distinct ways during the development of different visual subcircuits.

## Methods

### Mice

*Fmr1* knockout mice were generated and genotyped as previously described [19]. Mice were back-crossed for at least five generations and maintained on a C57BL/6 background. Only male mice were used for both experimental and control animals to avoid potential mosaicism associated with heterozygous females and to facilitate the use of littermates as controls. Mice were housed in a temperature and humidity**-**controlled room under standard 12/12-h light-dark cycle. After weaning, mice were housed in groups of 1–5 with same-sex siblings. All procedures were performed in accordance with, and approved by, the Children’s National Health System IACUC.

### In vivo electrophysiology

Recordings were performed as previously described [17]. Briefly, adult male mice (postnatal day 60 (P60)–P120) were anesthetized with isofluorane. The animal’s temperature was monitored and maintained at 37 °C through a feedback-controlled heating pad. Silicone oil was applied on the eyes to prevent drying. A craniotomy was performed on the right hemisphere ~ 0.5 mm lateral from the midline suture and between 1.5 to − 0.25 mm anterior from the lambda suture. A 16-channel silicone probe (Neuronexus) coated in DiI was lowered between 0.8 and 1.5 mm into the SC at a 35° angle and stabilized with agarose (1% in PBS). Electrical signals were amplified and filtered between 0.7 and 7 kHz, sampled at 25 kHz, and acquired using a System 3 workstation (Tucker-Davis Technologies). Individual units were identified by spike sorting methods using independent component analysis, as described previously [20].

### Visual stimuli and receptive field determination

Visual stimuli were generated using custom software (MATLAB), as described previously [20]. The monitor (52 × 29.5 cm, 60-Hz refresh rate) was placed 22 cm from the animal in front of the eye contralateral to the recording penetration, subtending ~ 90 × 70° of visual space. We utilized a flashing spot stimulus in which a white square subtending 5 × 5° of visual space was displayed for 500 ms at pseudorandom locations for five trials at each location. Following presentation, the screen remained blank for 500 ms before the next stimulus presentation. Thus, individual trials could be divided into two sub-stimuli to isolate the ON and OFF responses of each cell. Spontaneous firing was determined during additional blank stimuli, and the threshold was set as the mean spontaneous rate plus two standard deviations. The ON and OFF responses were determined by counting the number of spikes occurring in a 200-ms window starting at 50 ms after spot onset and offset, respectively. The total responses were determined using the spikes occurring in both the ON and OFF windows. A cell was considered responsive to either or both stimuli if the spike rate exceeded threshold in at least 40% of trials. Since we did not assume that receptive fields would be shaped such that they could be fit with a 2-D Gaussian, receptive field area was calculated as the number of squares to which an individual unit responded, as described previously [21]. The length of azimuth (*L*_A_) and elevation (*L*_E_) axes, calculated as the number of squares eliciting a response along the horizontal and vertical axes of the receptive field, respectively, was used to calculate the axes ratio (*L*_A_/*L*_E_). Area ratio was calculated using ON and OFF subfield areas (*A*_ON_/*A*_OFF_). The overlap index measures the degree of overlap between the ON and OFF subfields while taking into account the distance between their centers. It is calculated using the equation: OI = (*W*_1_ + *W*_2_ − *c*)/(*W*_1_ + *W*_2_ + *c*), where *W*_1_ and *W*_2_ are the half-widths of the subfields measured along the line joining the subfield centers, and *c* is the distance between the ON and OFF subfield centers [14]. The response ratio was calculated with the peak ON and OFF responses (*R*_on,max_ /*R*_off,max_).

To calculate directional and axial tuning, drifting gratings of 100% contrast at 12 different orientations (30° spacing) and six different spatial frequencies between 0.01 and 0.32 cpd (six logarithmic steps) were presented. A temporal frequency of 2 Hz was consistent for all the gratings. Each stimulus of given orientation and spatial frequency (or a blank condition) was presented for 1.5 s in a pseudorandom order for five trials, with an interstimulus interval of 0.5 s. The preferred direction (*θ*_pref_) and preferred spatial frequency were determined as those at which the mean response was greatest. Selectivity was described using two indices: (1) direction selectivity index (DSI) = (*R*_pref_ − *R*_opp_)/(*R*_pref_ + *R*_opp_), where *R*_pref_ was the response at *θ*_pref_ and *R*_opp_ at *θ*_pref_ + π and (2) axis selectivity index (ASI) = (*R*_pref_ − mean(*R*_orth_))/(*R*_pref_ + mean(*R*_orth_)). The tuning curves were fitted with a sum of two Gaussians centered at *θ*_pref_ and *θ*_pref_ + π using the nlinfit function in MATLAB, and the tuning width was calculated as the half-width at half-maximum of the fitted curve above the baseline [20]. A cell was considered direction selective (DS) with a DSI greater than 0.33 and an OSI greater than 0.33. A cell was considered axis selective (AS) with an OSI greater than 0.33 and a DSI less than 0.33.

### Axon tracing

Focal and bulk labeling of retinal ganglion cells (RGCs) was performed as described previously [22]. Briefly, adult mice (> P40) were deeply anesthetized by subcutaneous injection of ketamine/xylazine solution (100/10 mg/kg) and the eye elevated out of the orbital socket by applying gentle pressure to the head. For focal labeling, a small amount (~ 50 nL) of 1,1′-dioctadecyl-3,3,3′,3′-tetramethylindocarbocyanine perchlorate (DiI, ThermoFisher) (10% in dimethylformamide (DMF)) was pressure injected into the nasal or temporal regions of the retina using a pulled glass pipet attached to a Picospritzer III (Parker-Hannafin). For bulk labeling, ~ 500 nL of fluorescently conjugated cholera toxin subunit b (CTb-488 or CTb-555, ThermoFisher) (2 mg/mL in phosphate-buffered saline (PBS)) was pressure injected into the posterior eye chamber using a pulled glass pipet and Picospritzer III.

Focal labeling of V1 neurons for anterograde tracing to the SC and retrograde tracing to the dorsal lateral geniculate nucleus (dLGN) was performed as previously described [23]. Briefly, adult mice were deeply anesthetized by subcutaneous injection of ketamine/xylazine solution (100/10 mg/kg). For anterograde labeling of V1-SC projections, a focal craniotomy was made over V1 using a 25 ga needle, and ~ 50 nL of DiI was pressure injected with a pulled glass pipet attached to a Picospritzer III. For retrograde labeling of geniculocortical projections, anesthetized mice were head fixed in a stereotaxic apparatus with ear bars. A focal craniotomy was made over V1 with an Ideal Micro Drill (Stoelting) and a Hamilton syringe with 33 ga needle attached was lowered ~ 400 μm into the neuropil. Using a Quintessential Stereotaxic Injector (Stoelting), 100 μL of CTb-555 (10 mg/mL) was injected at a rate of 0.5 mL/min.

### Tissue processing and microscopy

Immediately after in vivo recording or 1 week following tracer injection, mice were euthanized and intracardially perfused with ice-cold PBS followed by 4% paraformaldehyde (PFA). The brains and eyes were dissected out and post-fixed in 4% PFA overnight or 30 min, respectively. For retinal DiI injections, the brains were briefly washed in PBS and the contralateral cortical hemisphere was removed and the underlying SC was imaged in whole mount. The brains were then embedded in 2% agarose and 150-μm sections were cut in the coronal plane with a Manual Slice Vibratome (World Precision Instruments) in order to image the dLGN. In addition, the retinas were dissected, flat mounted, and imaged to verify DiI injection site size and location. For other injections, the brains were briefly washed in PBS, embedded in 2% agarose and 150-μm sections were cut in the coronal (bulk retinal labeling, CTb in V1) or sagittal (DiI in V1) plane. All imaging was performed on a BX61 microscope equipped with an AxioCam HR digital camera (Olympus).

### Statistical analysis

All values are reported as mean ± SEM. The distributions of all data were first tested for normality of distribution using the D’Agostino & Pearson normality test. Normally distributed data were compared using the Student’s *t* test, while other distributions were compared using the Kolmogorov-Smirnov test (K-S test) or the median ranks were compared using a Mann-Whitney test. For comparison of baseline and peak firing rates of the same neuron, a Wilcoxon matched-pairs signed rank test was used. All statistical tests were evaluated at *α* = 5% probability of false positives. All analyses were performed with the statistical software package Prism (GraphPad).

## Results

### Visual receptive field size and shape

**Fig. 1:**
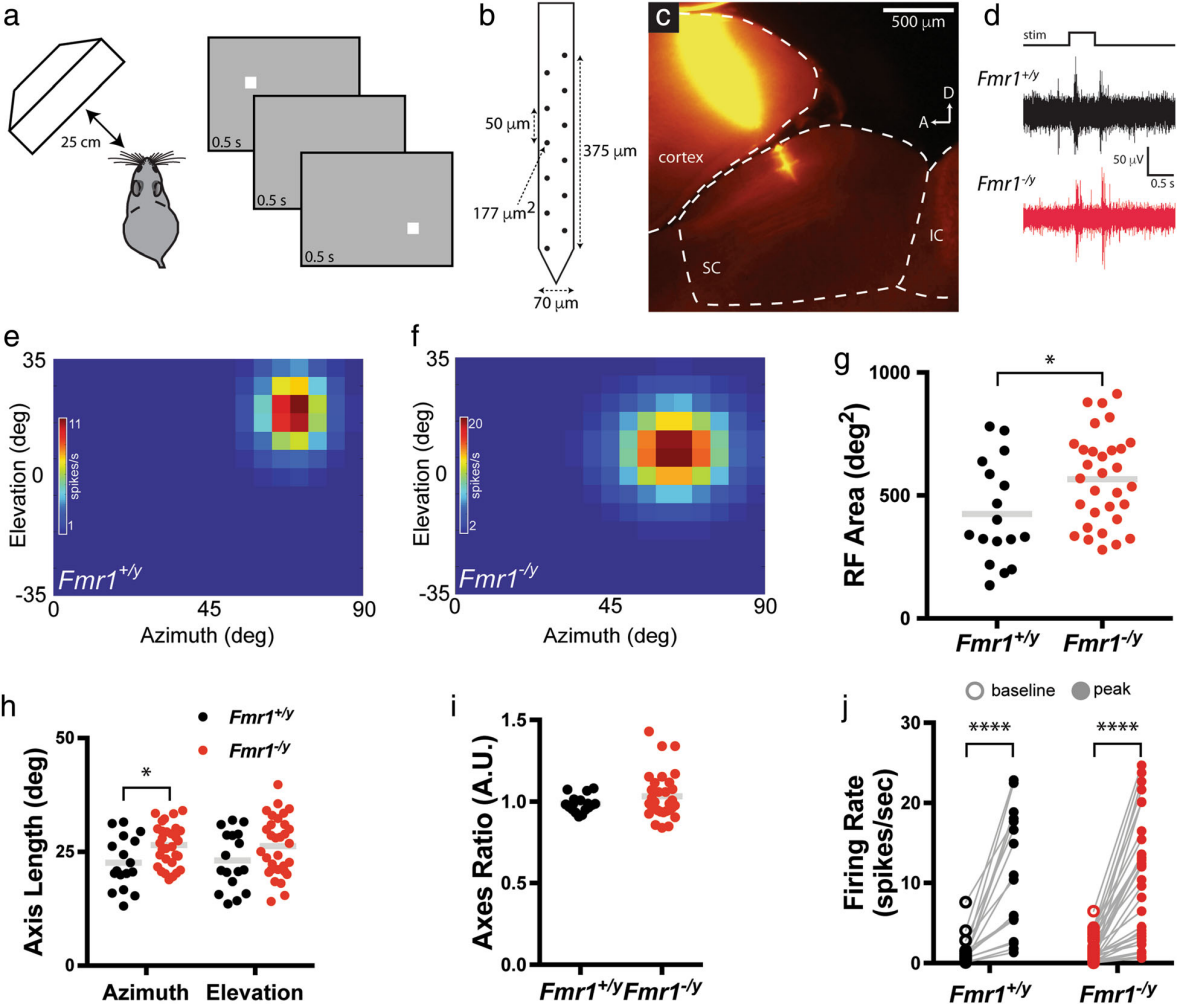
Enlarged receptive field size of visual neurons in the SC of *Fmr1*^*−/y*^ mice. **a** Schematic of in vivo visual stimulus presentation set up and flashing spot paradigm. **b** Schematic of the silicon multi-electrode array utilized. **c** Representative parasagittal section through the SC from a mouse in which recording was performed. The electrode was coated with fluorescent DiI in order to visualize the track and final position within the superficial layers of the SC. **d** Representative raw traces from recordings in the SC of *Fmr1*^*+/y*^ (black) and *Fmr1*^*−/y*^ (red) mice in response to the stimulus presented in **a**. **e**, **f** Heat maps of the total response to a flashing spot stimulus from representative visual neurons identified in the SC of *Fmr1*^*+/y*^ (**e**) and *Fmr1*^*−/y*^ (**f**) mice. **g** Quantification of the average receptive field (RF) area. *p < 0.05, unpaired *t* test. **h** Quantification of the length of the azimuth and elevation axes. *p < 0.05, unpaired *t* test. **i** Quantification of the azimuth to elevation axes ratio. **j** Quantification of the baseline and peak firing rates for individual neurons. ****p ≤ 0.0001, Wilcoxon matched-pairs signed rank test. For **g**–**j**, individual values for each identified neuron are shown, and the bar indicates the mean

To determine the receptive field properties of visual neurons in the SC of *Fmr1*^*−/y*^ mice, we recorded extracellular signals from the SC of isofluorane anesthetized mice while presenting computer-controlled visual stimuli. To begin, we presented mice with a flashing spot stimulus in which a white square occupying 5 × 5° of visual space was shown on gray background in a pseudorandom order (Fig. 1a). Individual units were identified post hoc and spikes associated with each unit were correlated with the stimulus presentation. Consistent with previous studies [14, 24], receptive field structures in the SC of *Fmr1*^*+/y*^ control mice were roughly circular in shape, with the most robust responses elicited by stimuli in the center of the receptive field (Fig. 1e & f). We found that receptive fields of visual neurons in the SC of *Fmr1*^*−/y*^ mice were qualitatively similar in structure to those of controls, though they appeared larger (Fig. 1e & f). Indeed, the mean area of receptive fields in *Fmr1*^*−/y*^ mice was significantly increased compared to those found in control mice (*Fmr1*^*+/y*^: 425.00 ± 50.13 deg^2^, *n* = 17 units from 1 to 3 penetrations each in 9 mice; *Fmr1*^*−/y*^: 566.30 ± 33.49 deg^2^, *n* = 31 units from 1 to 4 penetrations each in 11 mice; *p* = 0.0197, unpaired *t* test) (Fig. 1g). Interestingly, quantification of the extent of visual space monitored along the horizontal (azimuth) and vertical (elevation) axes suggested that the shape of receptive fields was slightly changed in knockouts, as we observed a significant increase in the mean length of the azimuth axis (*Fmr1*^*+/y*^: 22.59 ± 1.39°; *Fmr1*^*−/y*^: 26.49 ± 0.81°; *p* = 0.0124, unpaired *t* test), but this was not observed for the elevation axis (*Fmr1*^*+/y*^: 23.09 ± 1.54°; *Fmr1*^*−/y*^: 26.28 ± 1.16°; *p* = 0.1059, unpaired *t* test) (Fig. 1h). However, we observed no change in the average ratio of azimuth to elevation axes (*Fmr1*^*+/y*^: 0.9863 ± 0.01236; *Fmr1*^*−/y*^: 1.0340 ± 0.02557; *p* = 0.4576, K-S test) (Fig. 1f), suggesting that the shape of receptive fields were roughly the same for neurons in the SC of *Fmr1*^*−/y*^ mice, despite the increase in size.

### ON and OFF subfield size, shape, and organization

**Fig. 2:**
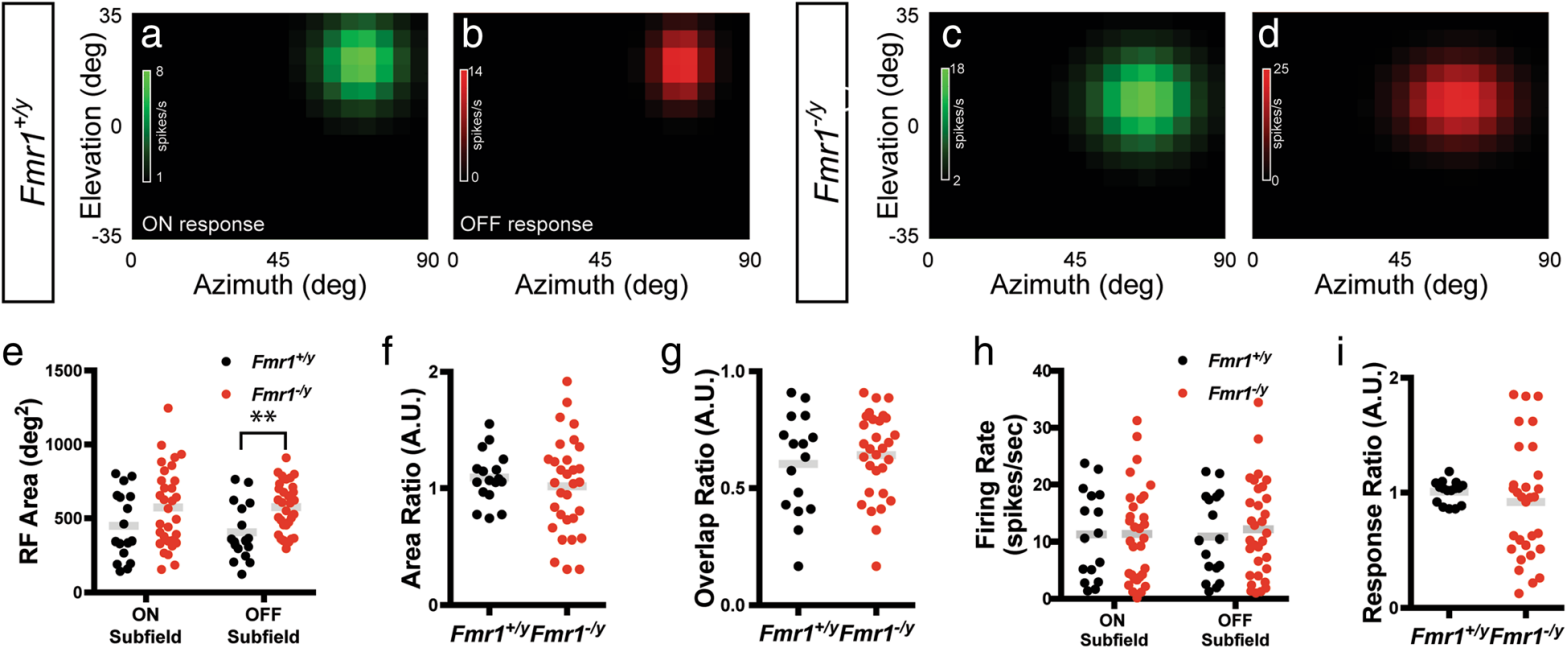
Enlarged OFF, but not ON, subfield of visual neurons in the SC of *Fmr1*^*−/y*^ mice. **a**–**d** Heat maps of the isolated ON (**a** and **c**) and OFF (**b** and **d**) responses to the flashing spot stimulus from the representative neurons identified in the SC of *Fmr1*^*+/y*^ (**a** and **b**) and *Fmr1*^*−/y*^ (**c** and **d**) mice. **e** Quantification of the average ON and OFF receptive field (RF) area. **p < 0.01, unpaired *t* test. **f** Quantification of the ON to OFF area ratio. **g** Quantification of the ratio of ON and OFF subfield overlap. **h** Quantification of the average evoked firing rate in the ON and OFF subfields. **i** Quantification of the ON to OFF subfield response ratio. For **e**–**i**, individual values for each identified neuron are shown, and the bar indicates the mean

Most neurons in the SC respond to both the onset and offset of light presented anywhere in their receptive field. To determine if there were specific changes in either the ON or OFF subfield of visual neurons in the SC of *Fmr1*^*−/y*^ mice, we segregated spikes into bins containing those occurring when the square was present (ON) and those occurring when the square had disappeared (OFF) from a given location (Fig. 1a). Consistent with previous data [14, 24], we found that most cells identified in the SC of both *Fmr1*^*+/y*^ and *Fmr1*^*−/y*^ mice had quantifiable ON and OFF subfields that were roughly circular in shape (Fig. 2a–d). Interestingly, we found that the mean area of the OFF subfield was significantly increased in the knockout group (*Fmr1*^*+/y*^: 404.1 ± 46.24 deg^2^; *Fmr1*^*−/y*^: 576.0 ± 30.16 deg^2^; *p* = 0.0023, unpaired *t* test), while the mean area of the ON subfield was unchanged (*Fmr1*^*+/y*^: 448.3 ± 56.02 deg^2^; *Fmr1*^*−/y*^: 573.4 ± 48.27 deg^2^; *p* = 0.1127, unpaired *t* test) (Fig. 2e). However, this was not reflected in a change the ON to OFF subfield area ratio (*Fmr1*^*+/y*^: 1.092 ± 0.05394 arbitrary units (A.U.); *Fmr1*^*−/y*^: 1.018 ± 0.07387 A.U.; *p* = 0.4972, unpaired *t* test) (Fig. 2f). Similarly, we observed no difference in the degree of overlap between ON and OFF subfields (*Fmr1*^*+/y*^: 0.603 ± 0.05394 A.U.; *Fmr1*^*−/y*^: 0.641 ± 0.03346 A.U.; *p* = 0.5364, unpaired *t* test) (Fig. 1g). Further, we observed no change in the ratio of the ON to OFF response rate (*Fmr1*^*+/y*^: 1.007 ± 0.02449 A.U.; *Fmr1*^*−/y*^: 0.919 ± 0.09185 A.U.; *p* = 0.4950, unpaired *t* test) (Fig. 2h). Together, these data suggest that the increased receptive field size of visual neurons in the SC of *Fmr1*^*−/y*^ mice may reflect a specific increase in the OFF subfield size, but that the general structure of receptive fields is not altered in the absence of *Fmr1*.

### Response to drifting square wave stimulus

**Fig. 3:**
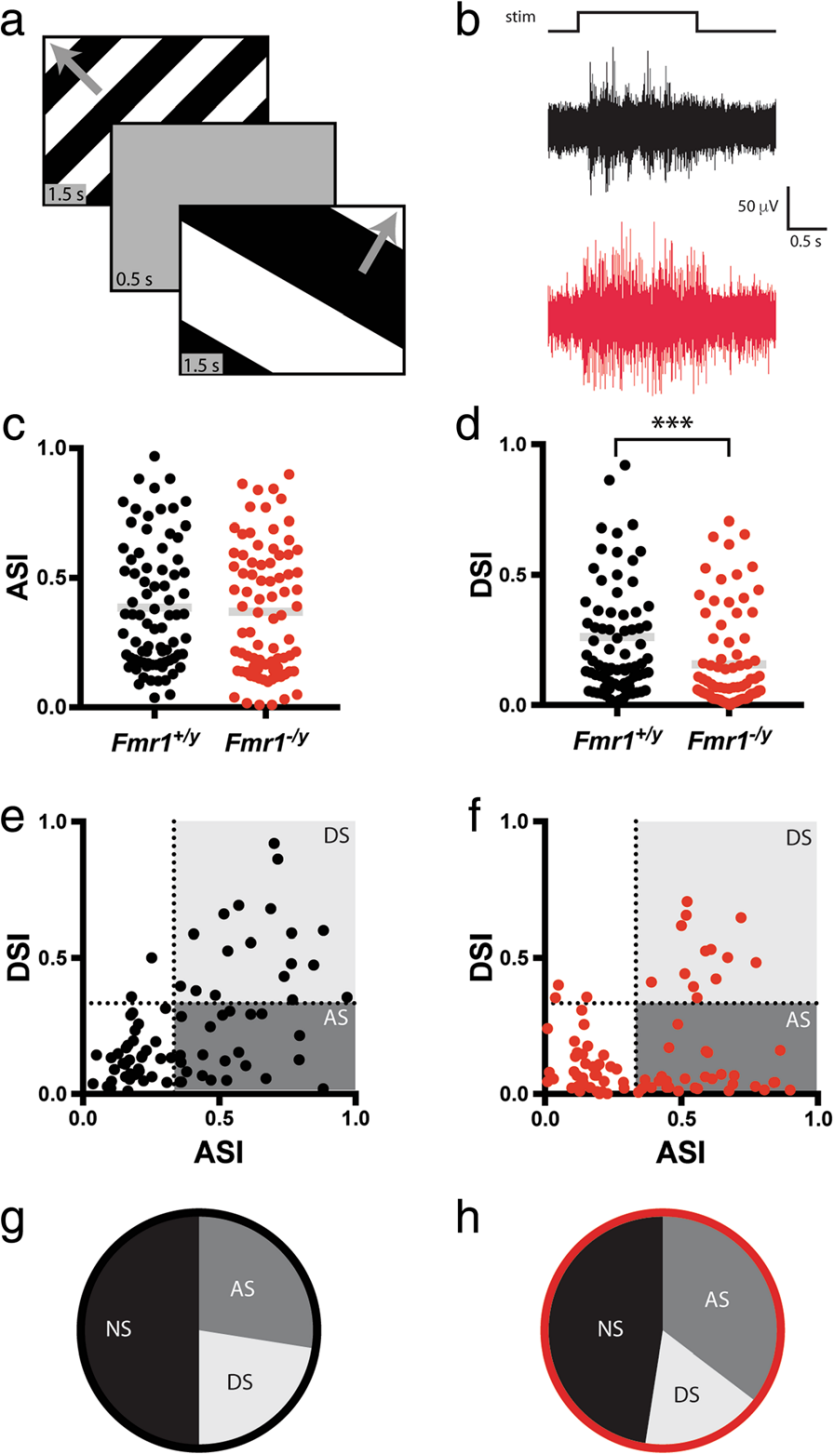
Decreased direction selectivity of visual neurons in the SC of *Fmr1*^*−/y*^ mice. **a** Schematic of drifting square wave visual stimulus paradigm. **b** Representative raw traces from recordings in the SC of *Fmr1*^*+/y*^ (black) and *Fmr1*^*−/y*^ (red) mice in response to the stimulus presented in **a**. **c**, **d** Quantification of the axis (**c**) and direction (**d**) selectivity indices (ASI, DSI) for all neurons identified in response to the drifting square wave stimulus. Individual values for each identified neuron are shown, and the bar indicates the mean. ***p < 0.001, Kolmogorov-Smirnov test. **e**, **f** Scatter plots of DSI and ASI for each identified neuron in the SC of *Fmr1*^*+/y*^ (**e**) and *Fmr1*^*−/y*^ (**f**) mice. Dotted lines at 0.33 indicate threshold for selectivity. Neurons classified as direction selective (DS) fall in the top right of the plot (*light gray shading*), while neurons classified as axis selective (AS) fall into the bottom right of the plot (*dark gray shading*). **g**, **h** Relative proportions of each type of neuron identified in the SC of *Fmr1*^*+/y*^ (**g**) and *Fmr1*^*−/y*^ (**h**) mice

We next asked if other aspects of visual function might be altered in the SC of *Fmr1*^*−/y*^ mice by using a drifting square wave stimulus (Fig. 3a), which allows the identification of direction-selective (DS) and axis-selective (AS) neurons, both of which are found in relative abundance in the SC. We presented square waves moving in 12 different orientations and at 6 different spatial frequencies. To begin, we calculated the axis- and direction selectivity indices (ASI, DSI), which measure the response rate at the preferred orientation compared to the orthogonal (ASI) or opposing (DSI) orientation. While we found no difference in the cumulative distribution of ASIs between groups (*Fmr1*^*+/y*^: 0.385 ± 0.02681, *n* = 80; *Fmr1*^*−/y*^: 0.369 ± 0.02716, *n* = 82; *p* = 0.2032, K-S test) (Fig. 3b), there was a significant shift in the cumulative distribution of DSIs for visual neurons in the SC of *Fmr1*^*−/y*^ mice towards less selectivity (*Fmr1*^*+/y*^: 0.2613 ± 0.02864; *Fmr1*^*−/y*^: 0.1559 ± 0.02017; *p* = 0.0004, K-S test) (Fig. 3c). By plotting the DSI as a function of ASI for each identified neuron, we were able to visualize the proportion of DS and AS neurons (Fig. 3d, e). Neurons classified as DS fall into the upper right corner of this plot, surpassing our threshold of 0.33 for both ASI and DSI; neurons classified as AS fall into the bottom right of this plot, surpassing a threshold of 0.33 for ASI, but falling below this for DSI. Using these criteria, we found no change in the overall number of neurons selective for some aspect of the drifting square wave stimulus (*Fmr1*^*+/y*^: 50.0%; *Fmr1*^*−/y*^: 52.3%), but we did observe a slight shift in the proportions of AS and DS neurons (*Fmr1*^*+/y*^: DS = 22.5%, AS = 27.5%; *Fmr1*^*−/y*^: DS = 17.1%, AS = 35.4%) (Fig. 3f, g); however, this change was not significantly different (Fisher’s exact test, two-tailed *p* = 0.2676). Together, these data suggest that visual neurons in the SC of *Fmr1*^*−/y*^ mice are less selective for direction of movement, but the proportion of DS neurons is not altered.

### Direction-selective neurons

**Fig. 4:**
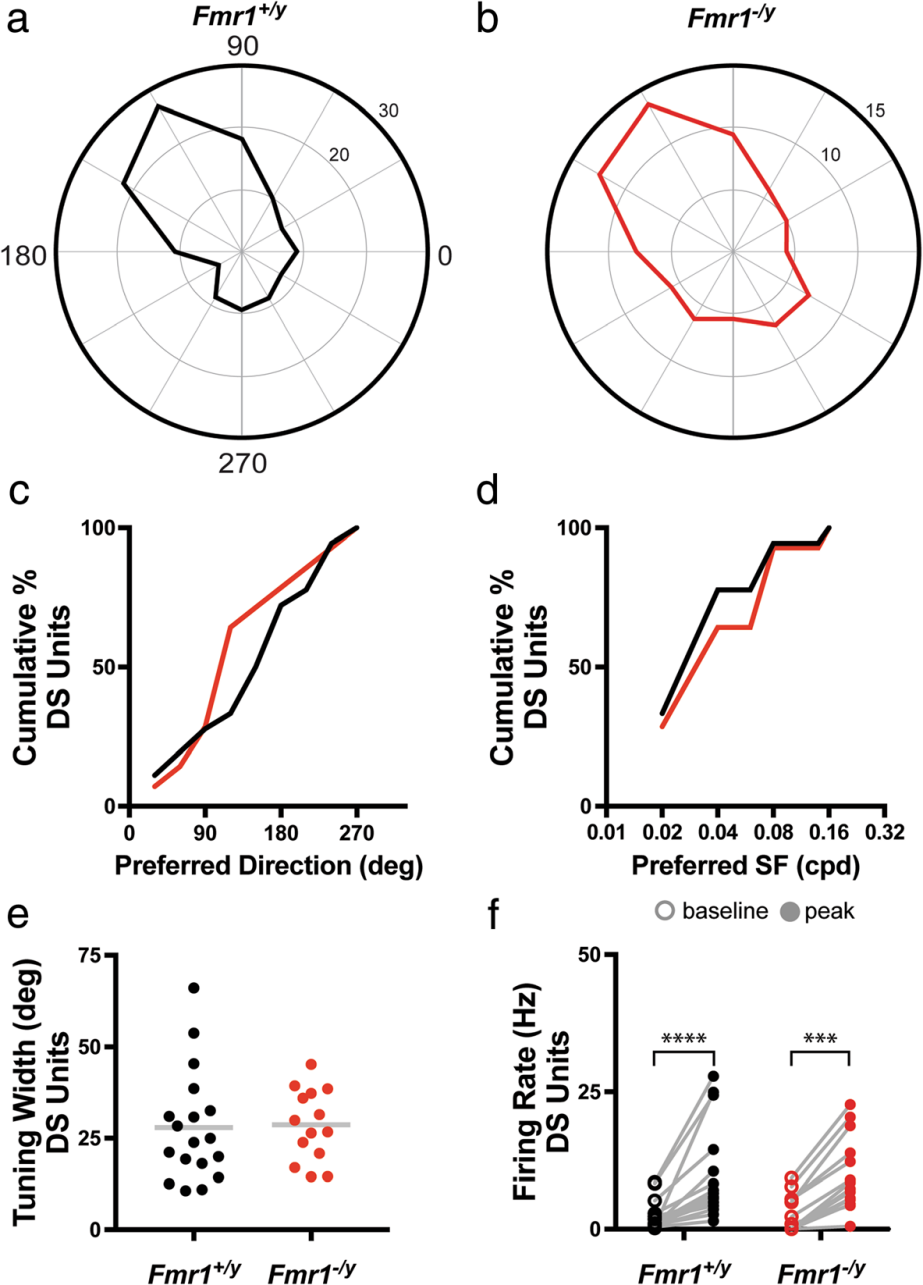
Properties of direction-selective neurons in the SC are unchanged in *Fmr1*^*−/y*^ mice. **a**, **b** Polar plots of representative direction-selective (DS) neurons in the SC of *Fmr1*^*+/y*^ (**a**) and *Fmr1*^*−/y*^ (**b**) mice. Orientation of the stimulus is represented around the circumference of the plot, and firing rate is indicated by concentric rings increasing centrifugally. **c** Quantification of the cumulative probability of DS neurons with different preferred directions. **d** Quantification of the cumulative probability of DS neurons with different preferred spatial frequencies. **e** Quantification of the tuning width of each DS neuron, bar indicates the mean. **f** Quantification of the baseline and peak firing rates for individual DS neurons. ****p ≤ 0.0001, Wilcoxon matched-pairs signed rank test

Based on the reduced DSI and slight reduction in proportion of DS neurons in the SC of *Fmr1*^*−/y*^ mice, we next wondered if other aspects of DS tuning might be affected due to loss of FMRP expression. Representative examples of the tuning curves of DS neurons from *Fmr1*^*+/y*^ and *Fmr1*^*−/y*^ SC are shown in Fig. 4a, b, which appear grossly similar. Consistent with this, we found no difference in the proportions of DS neurons exhibiting preference for different directions of movement (*p* = 0.4375, K-S test) nor different spatial frequencies (*p* = 0.9810, K-S test) (Fig. 4c, d), suggesting that tuning diversity of DS neurons is still intact in the absence of *Fmr1*. Additionally, the sharpness of tuning, measured by the average tuning width of DS neurons was unchanged (*Fmr1*^*+/y*^: 27.90 ± 3.575°, *n* = 18; *Fmr1*^*−/y*^: 28.68 ± 2.622°, *n* = 14; *p* = 0.8682, unpaired *t* test) (Fig. 4e). Finally, while we observed a significant difference in the peak firing rate compared to the baseline rate for DS neurons in both the *Fmr1*^*+/y*^ and *Fmr1*^*−/y*^ groups (*Fmr1*^*+/y*^: baseline = 2.283 ± 0.6782, peak = 9.833 ± 2.123, *p* < 0.0001, Wilcoxon matched-pairs signed rank test; *Fmr1*^*−/y*^: baseline = 2.6 ± 0.8846, peak = 9.994 ± 1.784, *p* < 0.0001, Wilcoxon matched-pairs signed rank test), there was no difference between either across groups (baseline, *p* = 0.3590, Mann-Whitney; peak, *p* = 0.6158, Mann-Whitney) (Fig. 4f). Together, these data suggest that despite an overall reduction in selectivity for direction of movement in the SC of *Fmr1*^*−/y*^ mice, the existing DS neurons are normally tuned.

### Axis-selective neurons

**Fig. 5:**
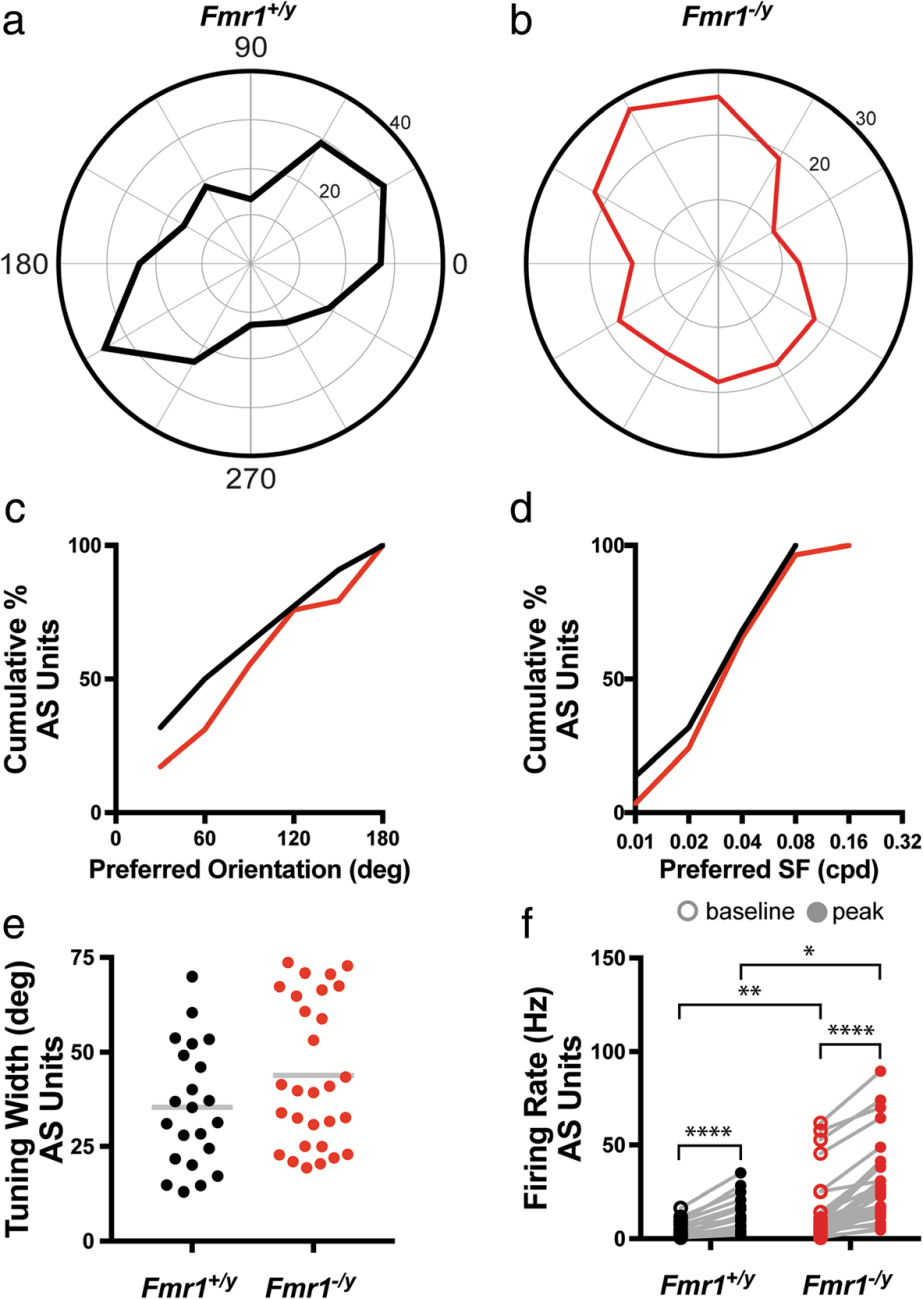
Increased baseline and peak firing rate of axis-selective neurons in the SC of *Fmr1*^*−/y*^ mice. **a**, **b** Polar plots of representative axis-selective (AS) neurons in the SC of *Fmr1*^*+/y*^ (**a**) and *Fmr1*^*−/y*^ (**b**) mice. Orientation of the stimulus is represented around the circumference of the plot, and firing rate is indicated by concentric rings increasing centrifugally. **c** Quantification of the cumulative probability of AS neurons with different preferred directions. **d** Quantification of the cumulative probability of AS neurons with different preferred spatial frequencies. **e** Quantification of the tuning width of each AS neuron, bar indicates the mean. **f** Quantification of the baseline and peak firing rates for individual AS neurons. *p < 0.05, Mann-Whitney test; **p < 0.01, Mann-Whitney test; ****p ≤ 0.0001, Wilcoxon matched-pairs signed rank test

We next evaluated the tuning properties of AS neurons in each group, representative examples of which are presented in Fig. 5a, b. Similar to what we found for DS neurons, we observed no change in the proportionality of AS neurons exhibiting preference for different axes of movement (*p* = 0.7591, K-S test) nor spatial frequencies (*p* = 0.9995, K-S test) (Fig. 5c, d). Additionally, we found no difference between groups in the cumulative distribution tuning widths for AS neurons (*Fmr1*^*+/y*^: 35.41 ± 3.439°, *n* = 22; *Fmr1*^*−/y*^: 43.85 ± 3.563°, *n* = 29; *p* = 0.3438, K-S test) (Fig. 5e), suggesting that the sharpness of tuning is similar between groups. As we observed for DS neurons, the peak rate of firing was significantly increased compared to the baseline rate for AS neurons in the SC of both *Fmr1*^*+/y*^ and *Fmr1*^*−/y*^ mice (*Fmr1*^*+/y*^: baseline = 5.776 ± 1.155, peak = 15.26 ± 2.677, *p* < 0.0001, Wilcoxon matched-pairs signed rank test; *Fmr1*^*−/y*^: baseline = 14.63 ± 3.229, peak = 29.01 ± 4.047, *p* < 0.0001, Wilcoxon matched-pairs signed rank test) (Fig. 5f). Intriguingly, we observed a significant increase in both the median baseline and median peak firing rate of AS neurons in the SC of *Fmr1*^*−/y*^ mice compared to AS neurons in control SC (baseline: *p* = 0.0271, Mann-Whitney; peak: *p* = 0.0063, Mann-Whitney) (Fig. 5f). Taken together, these data suggest that while the tuning properties of AS neurons in the SC of *Fmr1*^*−/y*^ mice are not substantially changed, they exhibit hyperexcitability compared to AS neurons in control SC.

**Fig. 6:**
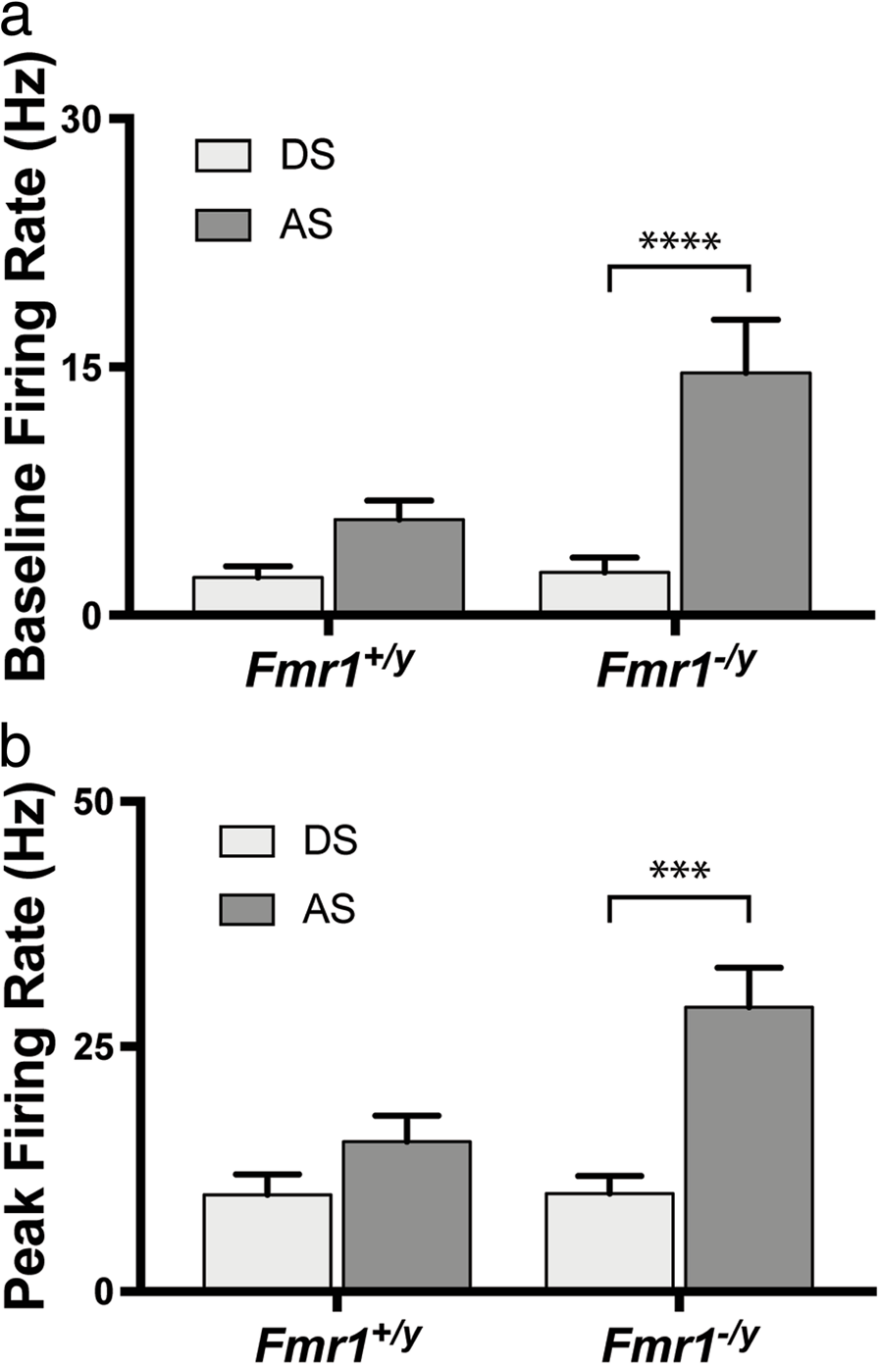
Comparison of baseline and peak firing rates of direction- and axis-selective neurons in the SC. **a**, **b** Quantification of the baseline (A) and peak (B) firing rates of direction-selective (*light gray*) and axis-selective (*dark-gray*) neurons in the SC of *Fmr1*^*+/y*^ and *Fmr1*^*−/y*^ mice. **p < 0.01, Kolmogorov-Smirnov test

Given that we found differences in the baseline and peak firing rates of AS, but not DS, neurons in the SC of *Fmr1*^*−/y*^ mice, we next wondered if AS neurons in the SC might be intrinsically more active in comparison to DS neurons. To do so, we compared the baseline and peak firing rates of DS and AS neurons in both control and *Fmr1*^*−/y*^ mice. In *Fmr1*^*+/y*^ mice, we found no difference in either the baseline or peak firing rates of DS and AS neurons (baseline: *p* = 0.1259, K-S test; peak: *p* = 0.2005, K-S test) (Fig. 6a, b). In contrast, we found that both the baseline and peak firing rates of AS neurons were significantly higher than those of DS neurons in the SC of *Fmr1*^*−/y*^ mice (baseline: *p* = 0.0034, K-S test; peak: *p* = 0.0036, K-S test) (Fig. 6a, b). Notably, the average baseline and peak rates of AS neurons were 5.6-fold and 2.9-fold greater than those of DS neurons, respectively. Together, these data suggest that in the absence of *Fmr1*, there is an AS circuit-specific increase in excitability of AS neurons, which is observed in both the spontaneous rate and visually evoked response.

### Organization of visual inputs to the SC

**Fig. 7:**
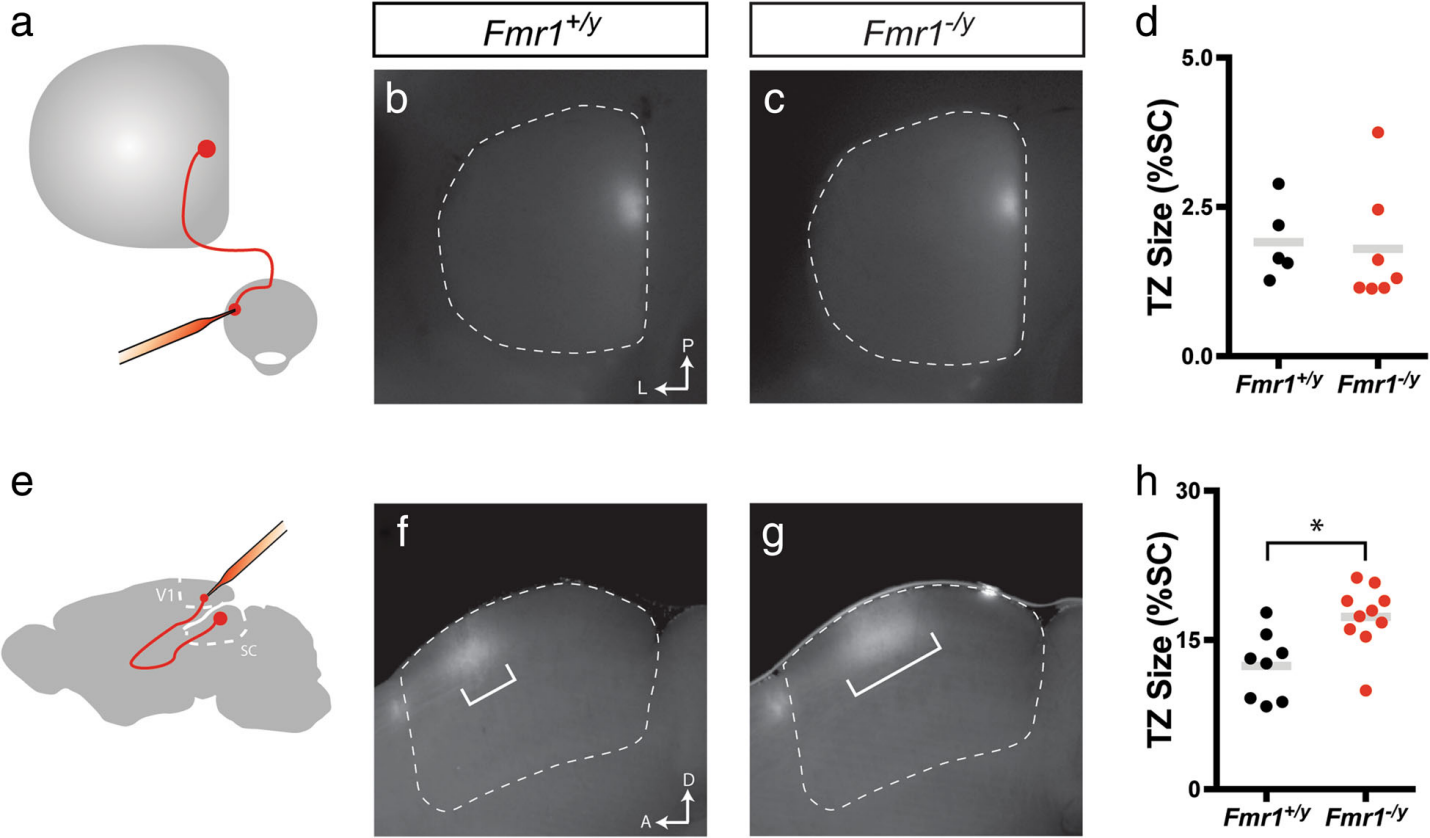
Altered corticocollicular topography in the SC of *Fmr1*^*−/y*^ mice. **a** Schematic of tracing paradigm to assess retinocollicular topography. **b**, **c** Whole mount images of the SC (dashed area) reveal the termination zones (TZs) of focally labeled retinal ganglion cells in *Fmr1*^*+/y*^ (**b**) and *Fmr1*^*−/y*^ (**c**) mice. **d** Quantification of the TZ size as a percent of the SC. **e** Schematic of tracing paradigm to assess corticocollicular topography. **f**, **g** Parasagittal sections through the SCs (dashed area) of *Fmr1*^*+/y*^ (**f**) and *Fmr1*^*−/y*^ (**g**) mice reveal the TZ of Layer 5 neurons labeled in the visual cortex. **h** Quantification of the TZ size as a percent of the SC. For **d** and **h**, individual values for each identified neuron are shown, and the bar indicates the mean. *p < 0.05, Kolmogorov-Smirnov test

Thus far, our in vivo electrophysiological data suggests that in *Fmr1*^*−/y*^ mice visual neurons in the SC have larger receptive fields, reduced direction selectivity, and alterations in the excitability of AS neurons. We next wondered if the anatomical organization of visual inputs to the SC might be disrupted in *Fmr1*^*−/y*^ mice. The SC receives visual inputs from retinal ganglion cells (RGCs) in the eye, as well as layer V neurons in visual cortical areas [11]. Each of these projections is organized topographically, such that neighbor-neighbor relationships of cell bodies are recapitulated in their axon terminals in the target. Further, layer V neurons in primary visual cortex (V1) terminate such that they are in topographic alignment with RGC inputs that monitor the same region of space. To begin to determine if *Fmr1* expression is required for proper targeting of visual inputs to the SC, we focally labeled small regions of the retina with the lipophillic tracer, DiI, which allows for visualization of the termination zones (TZs) of labeled RGC axons in the SC (Fig. 7a). Interestingly, we found that the TZs of retinal inputs in the SC of *Fmr1*^*−/y*^ mice were strikingly similar to those observed in control mice (Fig. 7b, c). Indeed, we found no difference in TZ size, measured as a percent of the SC, between groups (*Fmr1*^*+/y*^: 1.912 ± 0.286, *n* = 5 mice; *Fmr1*^*−/y*^: 1.796 ± 0.3715, *n* = 7 mice; *p* = 0.5455, K-S test) (Fig. 7d). We then traced projections from the primary visual cortex (V1) to the SC by focally injecting DiI into the cortices of adult mice and visualized TZs in sagittal sections (Fig. 7e). We found that V1-SC TZs in *Fmr1*^*−/y*^ mice were localized to the correct sublamina; however, they appeared larger than those found in control mice (Fig. 7f, g). Indeed, we found a significant increase in the TZ size, measured as a percent of the SC, in *Fmr1*^*−/y*^ mice (*Fmr1*^*+/y*^: 12.38 ± 1.206, *n* = 8 mice; *Fmr1*^*−/y*^: 17.33 ± 1.019, *n* = 10 mice; *p* = 0.0207, K-S test) (Fig. 7h). Together, these data demonstrate that retinal inputs to the SC appear unaffected in the absence of *Fmr1*, but that the refinement of cortical V1 inputs may be disrupted, indicating a possible misalignment with the retinal map.

**Fig. 8:**
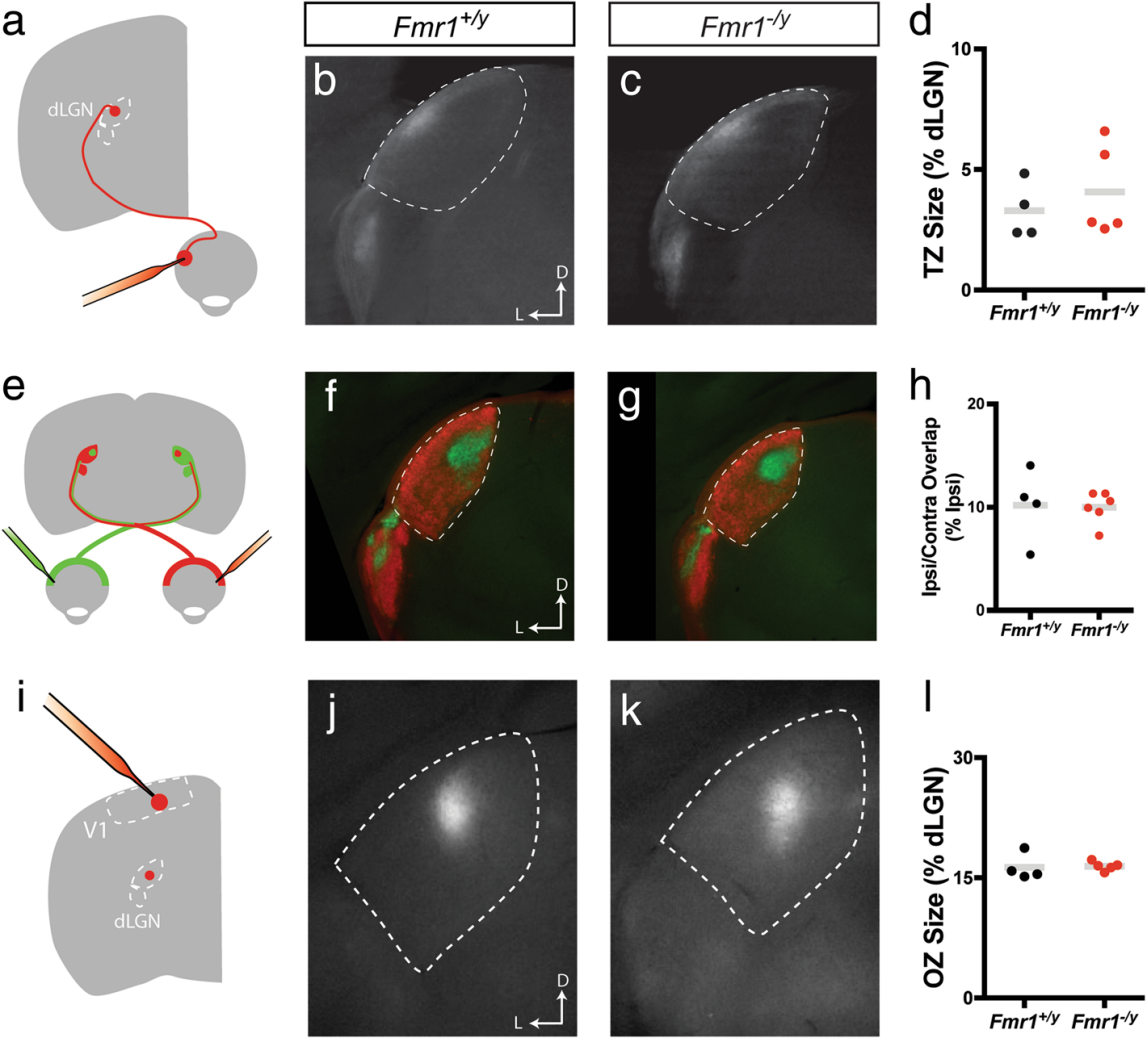
Topography of retinogeniculocortical pathway is preserved in *Fmr1*^*−/y*^ mice. **a** Schematic of tracing paradigm to assess retinogeniculate topography. **b**, **c** Coronal sections through the dorsal lateral geniculate nucleus (dLGN, dashed area) reveal the termination zones (TZs) of focally labeled retinal ganglion cells (RGCs) in *Fmr1*^*+/y*^ (**b**) and *Fmr1*^*−/y*^ (**c**) mice. **d** Quantification of the TZ size as a percent of the dLGN. **e** Schematic of tracing paradigm to assess eye-specific segregation in the dLGN. **f**, **g** Coronal sections through the dLGN (dashed area) reveal the terminations of bulk-labeled RGCs projecting from the contralateral (red) and ipsilateral (green) eye of *Fmr1*^*+/y*^ (**f**) and *Fmr1*^*−/y*^ (**g**) mice. **h** Quantification of the amount of overlap between ipsilateral and contralateral inputs in the dLGN, measured as a percent of the ipsilateral patch. **i** Schematic of tracing paradigm to assess geniculocortical topography. **j**, **k** Coronal sections through the dLGN (dashed area) reveal the origination zone (OZ) of geniculocortical neurons labeled in primary visual cortex of *Fmr1*^*+/y*^ (**l**) and *Fmr1*^*−/y*^ (**g**) mice. **h** Quantification of the OZ size as a percent of the dLGN. For **d**, **h**, and **l**, individual values for each mouse are plotted, and the bar indicates the mean

One potential explanation for the alteration in V1 inputs to the SC could be that they simply reflect topographic disorganization in the cortex. To test this possibility, we examined the anatomical organization of projections along the retino-geniculo-cortical pathway. We first examined RGC projections to the dorsal lateral geniculate nucleus (dLGN) by DiI tracing in adult mice (Fig. 8a). Similar to our findings for retinocollicular projections, we found that TZs in the dLGN appeared similar in both *Fmr1*^*+/y*^ and *Fmr1*^*−/y*^ mice (Fig. 8b, c). In fact, we observed no difference in TZ size, measured as a percent of the dLGN, between groups (*Fmr1*^*+/y*^: 3.283 ± 0.5857, *n* = 4 mice; *Fmr1*^*−/y*^: 4.067 ± 0.8487, *n* = 5 mice; *p* = 0.5635, K-S test) (Fig. 8d). To further investigate the organization of retinogeniculate projections, we assessed eye-specific segregation in the dLGN. To do so, we bulk-labeled RGCs by injecting fluorescently tagged cholera toxin subunit b of different colors (CTb-488 or CTb-555) in each eye and visualizing their terminals in the dLGN (Fig. 8e). Using this technique, we found that eye-specific segregation in the dLGN of *Fmr1*^*−/y*^ mice appeared similar to that of controls (Fig. 8f, g). Quantification of the overlapped pixels as a percent of the size of the ipsilateral patch confirmed this, as we found no difference between groups (*Fmr1*^*+/y*^: 10.21 ± 1.792, *n* = 4 mice; *Fmr1*^*−/y*^: 10.01 ± 0.6233, *n* = 6 mice; *p* = 0.9952, K-S test) (Fig. 8h). Finally, we assessed geniculocortical projection organization by focally injecting CTb-555 into V1 to retrogradely label neurons in the dLGN (Fig. 8i). Imaging of the origination zone (OZ) of labeled neurons revealed a similarly sized and shaped region in the dLGN of *Fmr1*^*+/y*^ and *Fmr1*^*−/y*^ mice (Fig. 8j, k). Quantification of OZ size also revealed no significant difference between groups (*Fmr1*^*+/y*^: 16.31 ± 0.8233, *n* = 4 mice; *Fmr1*^*−/y*^: 16.47 ± 0.2582, *n* = 5 mice; *p* = 0.4286, K-S test) (Fig. 8l). Taken together, these data suggest that the mature anatomical organization of projections along the retino-geniculo-cortical pathway are grossly normal in the absence of *Fmr1*. Combined with our retino- and corticocollicular tracing data, as well as our receptive field mapping, these data suggest that disruptions in V1 corticocollicular circuit formation may underlie deficits in visual function in the SC of *Fmr1*^*−/y*^ mice.

## Discussion

Deficits in sensory processing are commonly associated with many neurodevelopmental disorders including FXS. While psychophysical data suggests visual deficits in patients with FXS, as well as premutation carriers, a circuit-level understanding of visual dysfunction is lacking. Here, we demonstrate that visual circuit function and organization in a critical midbrain nucleus are disrupted in a mouse model of FXS. Specifically, visual neurons in the SC of *Fmr1*^*−/y*^ mice exhibit enlarged receptive field areas and reduced direction selectivity. In addition, we found that AS neurons specifically are hyperexcitable in the absence of FMRP, though their tuning properties are unaffected. Interestingly, our axon tracing data reveal that inputs to the SC from V1, but not the retina, are disrupted, having larger TZs than those found in control animals. Overall, these data argue that deficits in visual processing and development of connectivity are subcircuit specific and thus suggest that FMRP may perform distinct functions in the assembly of different circuits. Further, elucidation of these deficits in the SC, a nucleus critically involved in the generation of goal-directed eye movements, opens the possibility for future investigations of the SC as a novel therapeutic target in FXS.

### A novel role for FMRP in visual circuit formation in the SC

The development of properly organized receptive fields in the SC is likely due to a combination of molecular cues and activity-dependent forces that guide RGCs to the appropriate post-synaptic partners in the SC. Indeed, mutant mice lacking ephrin-A molecules involved in map formation exhibit misshapen receptive fields in the SC [21]. And, in mouse mutants in which the normal pattern of spontaneous retinal activity is disrupted (β2^−/−^), the receptive fields of neurons in the SC are increased and expanded along the azimuth axis [25]. Our findings in *Fmr1*^*−/y*^ mice are similar to these data, suggesting a role for FMRP in the activity-dependent formation of retinocollicular connectivity. Interestingly; however, we did not observe a disruption in retinocollicular topography, which is evident in β2^−/−^ mice [26], suggesting that the normal pattern of spontaneous retinal waves remain intact in *Fmr1*^*−/y*^ mice and that FMRP is not required for wave-dependent RGC axon terminal refinement and pruning. We did find that V1 corticocollicular projections were disrupted and potentially misaligned with the retinal map. Based on our previous work suggesting visual map alignment is dependent on cholinergic retinal waves [23], these data suggest that FMRP may play a specific role in wave-driven refinement of V1-SC corticocollicular arbors, but not in wave-driven refinement of RGC terminals.

Previous studies suggest that both RGCs and V1 corticocollicular neurons establish connections with common post-synaptic targets in the SC [27], so it is unclear why retinocollicular refinement is unaffected in *Fmr1*^*−/y*^ mice, but the refinement of V1-SC terminals is disrupted. Such specificity could be due to the regulation of FMRP expression temporally or spatially during development. Retinocollicular mapping occurs during the first postnatal week, while alignment of corticocollicular projections occurs in the second postnatal week [13]. Thus, FMRP expression in the SC could be temporally regulated: not expressed in the SC—and, thus, not required—during retinocollicular refinement, but expressed during V1-SC corticocollicular refinement to play a critical role in this process. Alternatively, FMRP expression could be restricted spatially, which could result in the differential effects of its loss on retinocollicular and corticocollicular refinement. That is, FMRP could be specifically expressed by V1 neurons projecting to the SC, but not in RGCs, during development. While there is evidence for FMRP expression in adult SC and retina [28, 29], a better understanding of the developmental timing of FMRP expression is needed in order to begin testing these possibilities.

### Altered direction selectivity in *Fmr1*^*−/y*^ mice

The nature of visual dysfunction in patients with FXS has been described as being specific to the magnocellular pathway [8], which in part processes information about object location and movement [30]. Intriguingly, we observed associated circuit-specific deficits in the SC of *Fmr1*^*−/y*^ mice, in that direction selectivity is reduced and receptive field size is increased, which combined could reduce the ability to localize objects and detect their movement. Interestingly, however, while we found a decrease in DSI, this was not reflected in a significant change in the proportion of DS neurons in the SC of *Fmr1*^*−/y*^ mice. One possible explanation for this discrepancy is that the remaining DS neurons are less selective, which could arise if DS neurons became more responsive to movement opposite to their preferred orientation. Another possibility is that this parameter of tuning is decreased in non-selective or AS neurons, perhaps due to the elevated basal and evoked firing rates we report here.

Our data suggest a reduction in direction selectivity in the SC of *Fmr1*^*−/y*^ mice, but the mechanism underlying this remains unclear. Recent studies in the mouse SC using distinct methodologies suggest that direction selectivity is inherited from DS RGCs [17, 18]. While our tracing data suggest that retinal projections to the SC are grossly normal, it remains possible that the specific connectivity patterns of DS RGCs are disrupted. Alternatively, the tuning and selectivity of DS RGCs could be affected, which is in turn relayed to SC neurons. Another possibility is that loss of FMRP expression results in a loss of DS neurons in the retina. Recent work suggests multiple changes in retinal function and synaptic protein expression; however, effects were primarily limited to the outer retina [31]. Future studies leveraging molecular markers of particular subtypes of RGCs and SC neurons are needed to determine if changes in tuning or loss of any specific cell type occurs in *Fmr1*^*−/y*^ mice.

### FMRP regulates the excitability, but not tuning, of axis-selective neurons

In many neurodevelopmental disorders, including FXS, alterations in excitatory-inhibitory balance have been reported in multiple circuits [32–35]. Intriguingly, we found that in the SC, alterations in excitability were restricted to AS neurons, while DS neurons and ON/OFF neurons in knockout SC showed similar spontaneous and evoked firing rates in comparison to controls. This subcircuit-specific change supports the hypothesis that FMRP performs distinct functions in a circuit-specific manner. But how might such a change occur? One possibility is an alteration in the number or strength of GABAergic inputs to AS neurons in the SC. In support of this, inhibitory neurons are densely packed into the SC [36] and the GABAergic transmission is dramatically altered in *Fmr1*^*−/y*^ mice [37]. Alternatively, the disrupted organization and, presumably, connectivity of V1 corticocollicular neurons in the SC could underlie the increase in firing rate. Interestingly, the spontaneous rate of neurons in the SC was increased in a previous study in which the cortex was removed [14], supporting this possible mechanism. Optogenetic and/or chemogenetic manipulation of corticocollicular inputs or local inhibitory neurons are needed to further test these possibilities.

Surprisingly, we found that despite the increased excitability of AS neurons in the SC of *Fmr1*^*−/y*^ mice, the tuning properties—such as preferred directions, spatial frequency preference, and sharpness of tuning—of these neurons was spared. Thus, within the SC, FMRP may regulate the gain of AS neuron firing rate, which is separable from the selectivity. Interestingly, the tuning and gain of response are also separable for neurons in the SC that are tuned to a looming stimulus [38]. In that study, activation of V1 reduced the gain of looming neurons, but other aspects of tuning, such as preferred speed, were unaffected. These findings are consistent with our data, in that we found a disorganization of V1 corticocollicular projections. Thus, a primary function of V1-SC inputs may be to modulate the gain of multiple, but not all, SC subcircuits. It would be interesting to test this hypothesis directly using optogenetic techniques.

## Conclusions

Here, we demonstrate different deficits in distinct visual subcircuits in the SC of a mouse model of FXS. These data suggest that FMRP may function in multiple ways during the assembly of sensory circuits. Further, our findings suggest that the SC may be an attractive model to further understand circuit dysfunction in the context of FXS and, potentially, be a novel target for therapeutic intervention.

## Abbreviations

AS: Axis selective
ASD: Autism spectrum disorder
ASI: Axis selectivity index
CTb: Cholera toxin subunit b
DiI: 1,1′-Dioctadecyl-3,3,3′,3′-tetramethylindocarbocyanine perchlorate
dLGN: Dorsal lateral geniculate nucleus
DS: Direction selective
DSI: Direction selectivity index
FMRP: Fragile X mental retardation protein
FXS: Fragile X syndrome
RGC: Retinal ganglion cell
SC: Superior colliculus
V1: Primary visual cortex
